# MDC and BLC are independently associated with the significant risk of early stage lung adenocarcinoma

**DOI:** 10.18632/oncotarget.13031

**Published:** 2016-11-03

**Authors:** Yanwei Zhang, Keke Yu, Song Hu, Yuqing Lou, Chunxing Liu, Jianlin Xu, Rong Li, Xueyan Zhang, Huimin Wang, Baohui Han

**Affiliations:** ^1^ Department of Pulmonary Medicine, Shanghai Chest Hospital, Shanghai Jiao Tong University, Shanghai, PR China; ^2^ Department of Biobank, Shanghai Chest Hospital, Shanghai Jiao Tong University, Shanghai, PR China; ^3^ Department of Research Center, Shanghai Chest Hospital, Shanghai Jiao Tong University, Shanghai, PR China; ^4^ Department of Laboratory Medicine, Huadong Sanatorium, Wuxi, Jiangsu Province, PR China

**Keywords:** early stage lung adenocarcinoma, subcentimeter lung adenocarcinoma, inflammatory biomarkers

## Abstract

**Background:**

This prospective study was designed to investigate the association between ten circulating inflammatory biomarkers and the risk for early stage lung adenocarcinoma.

**Methods:**

All inflammatory biomarkers were measured in 228 patients with early stage (IA to IIB) lung adenocarcinoma and 228 age-, sex- and smoking-matched healthy controls by using the Luminex bead-based assay.

**Results:**

Only two biomarkers were significantly associated with the risk of early stage lung adenocarcinoma after the Bonferroni correction: the multivariate odd ratio (OR) (95% confidence interval or CI) was 0.29 (0.16-0.53) for MDC and 4.17 (2.23-7.79) for BLC for the comparison of patients in the 4^th^ quartile with the 1^st^ quartile (both P<0.0001). When analysis was restricted to never smokers (196 patients/196 controls), MDC and BLC were still significantly associated with the risk of early stage lung adenocarcinoma (OR, 95% CI, P: 0.37, 0.21-0.66, P<0.0001 for MDC and 2.78, 1.48-5.22, P =0.001 for BLC). Furthermore, elevated BLC was associated with a 2.90-fold (95% CI: 1.03-8.17, P=0.037) increased risk of subcentimeter lung adenocarcinoma, and there was an increasing trend for BLC with the progression of subcentimeter lung adenocarcinoma.

**Conclusion:**

Our findings demonstrated that MDC and BLC were independently associated with the significant risk of early stage lung adenocarcinoma, even in non-smokers and in stage IA patients. BLC was further identified to play a carcinogenic role in the progression of lung adenocarcinoma.

## INTRODUCTION

Lung cancer is the leading cause of cancer-related death worldwide. As an aggressive histopathologic type of lung cancer, lung adenocarcinoma has recently aroused extensive concerns of scientific community [1, 2]. The dismal 5-year survival rate of lung cancer is mainly due to late-stage diagnosis for the majority of patients. In fact, the stage of lung cancer has a major impact on survival rate, as up to 65% of patients diagnosed with early stage lung cancer survived five years compared to less than 10% of those entering an advanced stage at diagnosis [3, 4]. Detecting lung adenocarcinoma at an early stage is thus vital to improve the prognosis and prolong the survival in clinical practice.

There is compelling evidence in medical literature for the diagnostic utility of low-dose computed tomography (LDCT) in patients with early stage lung cancer. A national lung screening trial or NLST conducted in 2011 has showed that LDCT screening can reduce lung cancer mortality by 20% [5]. As recommended by the United States Preventive Services Task Force guidelines, it is necessary to implement annual LDCT screening for individuals at high risk for lung cancer [6]. Using LDCT screening, the detection rate of patients with stage I lung cancer was 70%, which was exceedingly higher than that of 16% under routine care [7], which highlights the importance of LDCT as a practical diagnostic tool for lung cancer. However, a major problem facing global researchers currently is the high false-positive rate of LDCT screening, as recorded in the NLST study: nearly 96% of abnormal results were false-positive, which resulted in unnecessary subsequent diagnostic screening procedures and even complications from invasive steps [5]. It is therefore of timely importance to identify biomarkers to facilitate the diagnostic utility of LDCT during lung cancer screening and predict the risk of early stage lung cancer.

Chronic inflammation is well established as a hallmark in lung carcinogenesis [8–10]. Several lines of evidence have revealed that inflammatory biomarkers such as C-reactive protein (CRP) can predict the significant risk of lung cancer [11–16]. For instance, elevated CRP was observed to confer a more than two-fold increased risk of lung cancer [11]. However, most of previous evidence on this subject was based on retrospective studies mainly involving smokers, and whether the resultant association with lung cancer can be extrapolated to non-smokers remains an open question. It is widely recognized that smoking status has a major impact on the molecular pathogenesis of lung cancer [17]. Moreover, the association of inflammatory biomarkers with lung cancer risk was rarely reported in early stage patients, who are clinically valuable to help identify susceptibility biomarkers. To fill this gap in knowledge, we therefore designed a prospective study, seeking to investigate the association between circulating inflammatory biomarkers and the risk for early stage lung adenocarcinoma among 228 patients and 228 matched controls. In addition, 85.96% of patients were never smokers and 69.74% were diagnosed at stage IA, which renders us sufficient power in further stratified explorations. In the present study, 10 widely-evaluated inflammatory biomarkers were measured in all study participants, including CRP, interleukin 1 alpha (IL-1a), interleukin 1 beta (IL-1b), interleukin 6 (IL-6), IL-10, interferon-gamma (IFN-r), transforming growth factor alpha (TGF-a), macrophage-derived chemokine (MDC), B lymphocyte chemoattractant (BLC) and monokine induced by gamma interferon (MIG), and they were previously reported to be associated with lung cancer [11–16, 18, 19].

## RESULTS

### Baseline characteristics

This study included 228 patients with early stage lung adenocarcinoma and 228 age-, sex- and smoking-matched controls, and their demographic and clinicopathologic characteristics are presented in Table 1. The mean (standard deviation or SD) age of patients was 58.86 (9.69) years, and 61.40% of them were female patients (n=140). Never smokers accounted for 85.96% of patients (n=196). Of 228 patients, 159 (69.74%) were at stage IA, 19 (8.33%) at stage IB and 50 (21.93%) at stage II.

**Table 1:** Baseline characteristics of early stage lung adenocarcinoma patients and matched controls

Characteristics	Patients (N=228)	Controls (N=228)	P value
Age (years), mean (SD)	58.86 (9.69)	58.82 (9.68)	0.970
Sex, N (%)			
Female	140 (61.40)	140 (61.40)	1.000
Male	88 (38.60)	88 (38.60)	
Smoking status, N (%)			
Ever smokers	32 (14.04)	32 (14.04)	1.000
Never smokers	196 (85.96)	196 (85.96)	
TNM stage			
IA	159 (69.74)	-	
IB	19 (8.33)	-	
IIA	19 (8.33)	-	
IIB	31 (13.60)		
Biomarkers (pg/mL), mean (SD)			
CRP	2057507.3 (321510.9)	1738483.5 (238799.1)	0.230
CXCL13/BLC	50.8 (29.2)	44.4 (35.3)	0.072
CCL22 /MDC	90.0 (47.0)	155.7 (127.9)	<0.001
CXCL9 /MIG	27.8 (25.7)	35.2 (33.3)	0.008
IL-6	12.2 (7.5)	11.1 (6.4)	0.077

*Abbreviations*: SD, standard deviation; N (%), number (percentage). P was calculated by the t-test or the Mann-Whitney U test or the Chisq test where appropriate.

### Inflammatory biomarkers and lung adenocarcinoma risk

The detection rates of 10 inflammatory biomarkers in circulation are shown in Supplementary Table S1. Notably, the detection rate was 100% for CRP, BLC, MDC and MIG, respectively.

Four out of 10 inflammatory biomarkers were associated with the risk of early stage lung adenocarcinoma at a significance level of 5% (Table 2 and Supplementary Table S2). For the comparison of patients in the 4^th^ quartile with the 1^st^ quartile, the multivariate OR (95% CI) was 0.29 (0.16-0.53) for MDC (P<0.0001), 4.17 (2.23-7.79) for BLC (P<0.0001), 0.40 (0.22-0.74) for MIG (P=0.013) and 0.58 (0.37-0.92) for IL-10 (P=0.021). Two biomarkers, MDC and BLC, survived the Bonferroni correction for multiple comparisons (P<0.05/10, here 10 is the total number of examined biomarkers). Pearson correlation analysis revealed a weak correlation between MDC and BLC (r=-0.022, P=0.645) (Supplementary Table S3). To yield more information, the BLC/MDC ratio was created to examine their prediction for the risk of early stage lung adenocarcinoma, and as expected there was a nearly 10-fold increased risk for patients in the 4^th^ quartile relative to patients in the 1^st^ quartile (Table 2).

**Table 2:** Risk prediction of MDC and BLC for early stage lung adenocarcinoma

Biomarkers, pg/mL	Patients N(%)	Controls N(%)	OR (95% CI), P	P_trend_
MDC				
<60.9	72 (31.6)	42 (18.4)	1	<0.0001
60.9-93.9	68 (29.8)	46 (20.2)	0.76 (0.43-1.35), 0.360	
93.9-140.6	51 (22.4)	63 (27.6)	0.40 (0.22-0.72), 0.002	
>140.6	37 (16.2)	77 (33.8)	0.29 (0.16-0.53), 5.7×10^−5^	
BLC				
<26.4	46 (20.2)	69 (30.3)	1	<0.0001
26.4-39.8	50 (21.9)	63 (27.6)	1.51 (0.86-2.67), 0.154	
39.8-57.0	58 (25.4)	58 (25.4)	1.77 (1.03-3.03), 0.038	
>57.0	74 (32.5)	38 (16.7)	4.17 (2.23-7.79), 8.0×10^−6^	
BLC/MDC				
1	23 (10.1)	91 (39.9)	1	<0.0001
2	62 (27.2)	52 (22.8)	5.46 (2.55-11.66), 1.2×10^−5^	
3	69 (30.3)	45 (19.7)	9.39 (4.26-20.72), 2.9×10^−8^	
4	74 (32.5)	40 (17.5)	9.98 (4.57-21.82), 8.2×10^−9^	

*Abbreviations*: N (%), number (percentage); OR, odds ration; 95% CI, 95% confidence interval. Adjusted for matching variables (age, sex and smoking history), history of chronic bronchitis/emphysema, history of coronary heart disease or heart attack, family history of lung cancer and regular use of aspirin/ibuprofen.

### Stratified analysis of significant inflammatory biomarkers

There were 196 patients with early stage lung adenocarcinoma and 196 matched controls who were both never smokers. When analysis was restricted to these never smokers, MDC and BLC were still significantly associated with lung adenocarcinoma risk, with the comparison of patients in the 4^th^ quartile with the 1^st^ quartile yielding an OR of 0.37 (95% CI: 0.21-0.66, P<0.0001) and 2.78 (95% CI: 1.48-5.22, P=0.001), respectively (Table 3). In addition, significance persisted after restricting analysis to 159 patients with stage IA lung adenocarcinoma and 159 matched controls, that is, elevated levels of MDC and BLC were associated with a 74% reduced (OR=0.26, 95% CI: 0.12-0.50, P<0.0001) and 110% increased (OR=2.10, 95% CI: 1.06-4.00, P=0.019) risk for stage IA lung adenocarcinoma, respectively (Table 3).

**Table 3:** Stratified analysis of MDC and BLC with early stage lung adenocarcinoma

No-smoking lung cancer risk					Stage IA lung cancer risk				
Biomarkers, pg/mL	Patients N (%)	Controls N (%)	OR (95% CI), P	P_trend_	Biomarkers, pg/mL	Patients N(%)	Controls N(%)	OR (95% CI), P	P_trend_
MDC					MDC				
<60.2	60 (30.6)	37 (18.9)	1	<0.0001	<60.3	50 (31.4)	29 (18.2)	1	<0.0001
60.2-92.1	60 (30.6)	39 (19.9)	1.00 (0.55-1.82), 0.990		60.3-91.9	48 (30.2)	32 (20.1)	0.87 (0.45-1.70), 0.690	
92.1-139.2	41 (20.9)	57 (29.1)	0.48 (0.26-0.86), 0.014		91.9-134.7	37 (23.3)	43 (27.0)	0.49 (0.25-0.98), 0.043	
>139.2	35 (17.9)	63 (32.1)	0.37 (0.21-0.66), 0.001		>134.7	24 (15.1)	55 (34.6)	0.26 (0.12-0.50), 0.00011	
BLC					BLC				
<26.4	41 (20.9)	56 (28.6)	1	0.001	<27.0	37 (23.3)	42 (26.4)	1	0.019
26.4-39.7	40 (20.5)	54 (27.6)	1.05 (0.89-1.87), 0.870		27.0-39.9	32 (20.1)	48 (30.2)	0.74 (0.52-1.40), 0.360	
39.7-55.7	51 (26.0)	51 (26.0)	1.60 (1.06-2.22), 0.230		39.9-56.9	40 (25.2)	40 (25.2)	1.21 (0.76-2.20), 0.120	
>55.7	64 (32.0)	35 (17.9)	2.78 (1.48-5.22), 0.001		>56.9	50 (31.4)	29 (18.2)	2.10 (1.06-4.00), 0.032	

*Abbreviations*: N (%), number (percentage); OR, odds ration; 95% CI, 95% confidence interval. Adjusted for matching variables (age, sex and smoking history), history of chronic bronchitis/emphysema, history of coronary heart disease or heart attack, family history of lung cancer and regular use of aspirin/ibuprofen.

### Inflammatory biomarkers and subcentimeter lung adenocarcinoma

**Figure 1:**
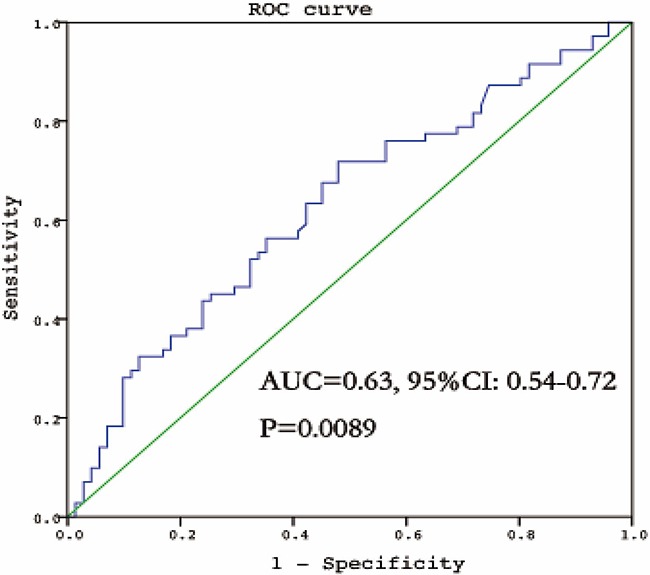
The ROC curve of BLC for the diagnosis of subcentimeter lung adenocarcinoma ROC, receiver operating characteristic; AUC, area under curve.

As shown in Table 4, BLC was associated with a 2.90-fold (95% CI: 1.03-8.17, P=0.037) increased risk of subcentimeter lung adenocarcinoma (tumor size less than 1cm) for the comparison of patients in the 4^th^ quartile with the 1^st^ quartile. Further ROC curve analysis revealed that BLC was a significant diagnostic biomarker for subcentimeter lung adenocarcinoma, with the area under the curve of 0.63 (95% CI: 0.54-0.72, P=0.0089) for BLC (Figure 1). The sensitivity, specificity and positive likelihood ratio were 0.72, 0.52 and 1.50, respectively (Supplementary Table S4).

**Table 4:** Risk prediction of MDC and BLC for subcentimetre lung adenocarcinoma

Biomarkers, pg/mL	Patients N (%)	Controls N (%)	OR (95% CI), P	P_trend_
MDC				
<60.5	20 (28.2)	15 (21.1)	1	0.058
60.5-91.7	21 (29.6)	15 (21.1)	1.09 (0.42-2.85), 0.86	
91.7-133.2	17 (23.9)	19 (26.8)	0.68 (0.27-1.73), 0.42	
>133.2	13 (18.3)	22 (31.0)	0.37 (0.14-1.11), 0.078	
BLC				
<26.2	15 (21.1)	20 (28.2)	1	0.037
26.2-41.8	15 (21.1)	22 (31.0)	0.90 (0.40-2.18), 0.79	
41.8-57.0	18 (25.4)	17 (23.9)	1.50 (0.62-3.55), 0.34	
>57.0	23 (32.4)	12 (16.9)	2.90 (1.03-8.17), 0.040	

*Abbreviations*: N (%), number (percentage); OR, odds ration; 95% CI, 95% confidence interval. Adjusted for matching variables (age, sex and smoking history), history of chronic bronchitis/emphysema, history of coronary heart disease or heart attack, family history of lung cancer and regular use of aspirin/ibuprofen.

**Figure 2:**
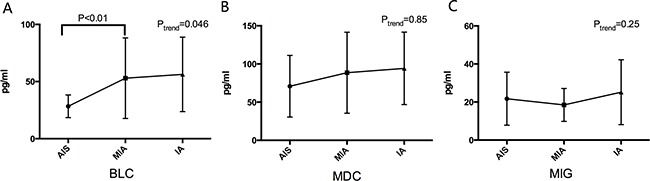
The different levels of BLC **A.** MDC **B.** and MIG **C.** among patients with AIS, MIA and IA. BLC, B lymphocyte chemoattractant, MDC, Macrophage-Derived Chemokine; MIG, Monokine Induced by Gamma Interferon; AIS, adenocarcinoma in situ; MIA, minimally invasive adenocarcinoma; IA, invasive adenocarcinoma.

The differences in inflammatory biomarkers among patients with adenocarcinoma in situ (AIS, pure lepidic growth), minimally invasive adenocarcinoma (MIA, predominant lepidic growth with invasion of 5 mm or less) and invasive adenocarcinoma (IA, predominant lepidic growth with invasion > 5 mm and other pathologic types of lung adenocarcinoma) showed that there was an increasing trend for BLC with the progression of subcentimeter lung adenocarcinoma from AIS to MIA and IA (P=0.046), as illustrated in Figure 2 and Supplementary Table S5.

## DISCUSSION

To the best of our knowledge, this is so far the first study that has prospectively evaluated multiple inflammatory biomarkers in circulation and the risk for early stage lung adenocarcinoma in a Chinese population. The key finding of this study was that two inflammatory biomarkers, MDC and BLC, were independently associated with the significant risk of early stage lung adenocarcinoma in overall population as well as in non-smokers and in stage IA patients. Moreover, BLC was found for the first time to have a close link with subcentimeter lung cancer risk, and this biomarker was proposed to play a carcinogenic role in the development and progression of lung adenocarcinoma.

Several large studies have showed that some inflammatory biomarkers could predict the risk of experiencing lung cancer [11–16, 20]. A prospective study by Shiels et al evaluated the relationship between 68 inflammatory biomarkers and lung cancer risk in 526 lung cancer patients and 592 matched controls, and 11 biomarkers were identified to be in significant association with lung cancer [11]. Subsequent replication of this association among the same lung cancer patients and enlarged matched controls (n=625) found that only 4 biomarkers remained statistically significant [20]. Although a large number of previous studies examined the susceptibility of inflammatory biomarkers to lung cancer risk, none have focused on patients with early stage lung cancer, as these patients are difficult to detect but have optimal survival benefits. To fill this void, we measured 10 widely-evaluated inflammatory biomarkers among 228 patients with early stage lung adenocarcinoma and 228 matched healthy controls, and our findings demonstrated that BLC and MDC were two significant independent predictors for the risk of experiencing early stage lung adenocarcinoma.

BLC, also known as B cell-attracting chemokine 1 (BCA-1) or Chemokine CXC ligand 13 protein (CXCL13), is a member of the CXC subtype of chemokine superfamily. As evidenced, BLC was involved in the carcinogenesis of several solid tumors, including breast cancer, neuronal cancer and prostatic cancer [21–24]. In addition, elevated BLC levels were observed in both non-small cell lung cancer (NSCLC) tissues and sera relative to controls [25]. Extending the results of previous studies, our findings demonstrated that BLC was a remarkable predictive factor for early stage lung adenocarcinoma, and patients in 4^th^ quartile of BLC levels were over 4 times more likely of experiencing early stage lung adenocarcinoma relative to those in 1^st^ quartile, consistent with the findings in the study by Shiels et al [11]. What's more, our findings also indicated a carcinogenic role of BLC in the progression of lung adenocarcinoma from AIS to MIA and IA. The biological mechanisms underlying this role are not fully understood, and further functional characterization is encouraged.

MDC, also known as C-C motif chemokine 22 (CCL22), is a member of the C-C family of chemokines that are produced by monocyte-derived macrophages and dendritic cells [26]. There is evidence that MDC was up-regulated in some tumor entities, suggesting its oncogenic potentials [27, 28]. The down-regulation of MDC in turn suggested its tumor suppressive potentials [29]. Nakanishi et al have found that elevated MDC expression was closely linked to longer disease-free survival time and lower risk of lung cancer recurrence after surgical resection [30]. Contrastingly in the study by Shiels et al, MDC was identified as a significant risk factor for lung cancer, that is, patients with elevated MDC levels had a 1.63-fold increased lung cancer risk in discovery phase and a 2.09-folded increased risk in replication phase [11]. Our findings instead supported a protective role of MDC in lung carcinogenesis. The reasons for this discrepancy are likely due to the study populations of different races (Chinese and Caucasian) and the confounding impact of smoking status between the present study and the study by Shiels et al [11]. In fact, over 90% of lung cancer patients were smokers in the study by Shiels et al [11], compared to only 14.4% in the present study.

CRP, a systemic marker of chronic inflammation, has been found correlated with increased lung cancer risk in many previous studies [11–15]. For instance, Chaturvedi et al found that elevated CRP levels are associated with nearly two-fold increased lung cancer risk [12]. However, in the present study, CRP levels did not show significant differences between the case and control subjects. The main cause maybe contributes to the intimate connection between cigarette smoking and evaluated CRP levels [31], since smokers were only accounted for 14.04% in our study.

It is generally recognized that the diagnosis of subcentimeter lung cancer is a challenging task for oncologists because of its tiny tumor size. Several studies have shown that lung cancer of 1 cm or less in diameter also has the potentials to spread locally, regionally and systemically [32, 33]. Thus, early detection of subcentimeter lung cancer is still a matter of contention. In this study, we interestingly found that patients with elevated BLC levels had nearly three-fold increased subcentimeter lung adenocarcinoma risk, and its sensitivity for diagnosing subcentimeter lung adenocarcinoma reached 72.0% yet the specificity is not that satisfactory (52.0%). Nevertheless, our findings provide a promising inflammatory biomarker for the prediction of subcentimeter lung adenocarcinoma, and we agree that further efforts are needed to combine this inflammatory biomarker with CT detection for the differential diagnosis of subcentimeter lung cancer as a whole.

Our study is not without limitations. On the one hand, our findings need to be replicated in another independent population to further confirm the predictive role of MDC and BLC in susceptibility to early stage lung adenocarcinoma. On the other hand, only lung andenocarcinoma patients were enrolled in the present study, and future studies with large samples specifically designed to examine the association of the two inflammatory biomarkers with lung squamous carcinoma and the other types of NSCLC are warranted.

In conclusion, our findings for the first time demonstrate that two inflammatory biomarkers, MDC and BLC, were independently associated with the significant risk of early stage lung adenocarcinoma, and this significant association persisted even in non-smokers and in stage IA patients. Moreover, BLC could be a promising biomarker for accessorily diagnosing subcentimeter lung cancer and may play a carcinogenic role in the progression of lung adenocarcinoma. For practical reasons, our findings may provide the basis for future personalized medicine whereby early stage lung cancer patients with abnormal inflammatory profiles can be detected earlier and receive timely therapeutic interventions.

## MATERIALS AND METHODS

### Study population

Lung cancer patients who were histologically confirmed according to the World Health Organization classification criteria [34] received lung resection in the Shanghai Chest Hospital between September 2013 and March 2015. The inclusion criteria of cases were: 1) patients with early stage (IA to IIB) lung adenocarcinoma; 2) available blood samples before lung resection. Reasons for exclusion were missing data on smoking history or family cancer history or missing consent for etiologic studies. Healthy controls were enrolled from Huadong Sanatorium, and one control subject was selected for each patient matched on age (±2 year), sex and smoking history. All study participants gave written informed consent before interview procedures and biospecimen collection. The conduct of this study was approved by the Institutional Review Boards of local hospitals.

From each patient, information was recorded at the time of enrollment, including age, sex, smoking history, family cancer history, medical history and working history. Pathologic stage was determined by the International Association for the Study of Lung Cancer (IASLC) TNM (tumor-node-metastasis) classification, 8^th^ edition [35].

### Inflammatory biomarkers

Preoperative blood samples were collected in coded heparinized tubes, and they were centrifuged at 2400-3000 rpm for 15 minutes at room temperature. Serum samples were frozen within 2 hours of collection and stored at −80°C until assayed.

On the basis of the results of previous studies [11, 15, 20], a total of 10 inflammatory biomarkers with potential implications in lung carcinogenesis were selected for measurement using the Luminex bead-based assay. Concentrations of 10 inflammatory biomarkers in circulation were calculated using a four or five parameter standard curve. All serum samples were assayed in duplicate and the results were averaged for analysis. Samples of both patients and matched controls were assayed on the same batch, and each patient-control pair was plated into adjacent wells. Within each batch, one pair of blinded duplicates and a pooled serum sample across batches were assayed to evaluate the reproducibility and drift across batches.

### Statistical analysis

Data were analyzed by the SPSS software version 20.0 (SPSS Inc., Chicago, IL, USA). Statistical significance was taken as two-sided P < 0.05, and multiple testing was controlled by the Bonferroni correction (P < 0.05 / 10 biomarkers).

For inflammatory biomarkers, measurements below the lowest limit of detection (LLOD) were assigned a value of half the LLOD as previously described [11]. All biomarkers were categorized into groups based on the proportion of individuals with measurements more than the LLOD as follows: biomarkers with more than 75% of individuals over the LLOD were categorized into quartiles; biomarkers with 50% to 75% of individuals over the LLOD were categorized into tertiles; biomarkers with 25% to 50% of individuals over the LLOD were categorized as below and above the median; and biomarkers with 10% to 25% of individuals over the LLOD were categorized as the undetectable and the detectable.

Conditional Logistic regression was used to quantify the association of inflammatory biomarkers with lung cancer risk, and effect-size estimates were expressed as odds ratios (ORs) and 95% confidence intervals (CIs). Besides the matched variables (age, sex and smoking history), adjustment also included history of chronic bronchitis/emphysema, history of coronary heart disease or heart attack, family history of lung cancer and regular use of aspirin/ibuprofen. In addition, the association of inflammatory biomarkers with lung cancer risk in non-smokers, in stage IA patients and in patients with subcentimeter tumor was separately analyzed. The relationship of inflammatory biomarkers was assessed by Pearson correlation analysis. Receiver operating characteristic (ROC) curve was created to evaluate the role of inflammatory biomarkers in diagnosing subcentimeter lung cancer. One-way ANOVA was used to compare inflammatory biomarkers among patients with adenocarcinoma in situ (AIS), minimally invasive adenocarcinoma (MIA) and invasive adenocarcinoma (IA).

## SUPPLEMENTARY TABLES


