# Supplementary material for: MDC and BLC are independently associated with the significant risk of early stage lung adenocarcinoma
Source: Oncotarget. 2016 Nov 3;7(50):83051–9. doi: 10.18632/oncotarget.13031 (PMC5347752; doi:10.18632/oncotarget.13031)
Supplement: Supplementary file 1 [file oncotarget-07-83051-s001.pdf]

MDC and BLC are independently associated with the significant risk of early stage lung adenocarcinoma

SUPPLEMENTARY TABLES

Supplementary Table S1: The detection rate of 10 inflammatory biomarkers

Inflammatory biomarkers	Detection rate
CRP	100.0%
BLC	100.0%
MDC	100.0%
MIG	100.0%
IL-6	60.3%
IFN-r	33.1%
IL1-b	25.0%
Il-10	20.6%
TGF-a	20.0%
IL1-a	20.0%

Supplementary Table S2: Risk prediction of other 8 inflammatory biomarkers for early stage lung adenocarcinoma

Biomarkers, pg/mL	Patients N(%)	Controls N(%)	OR (95% CI), P	P_{trend}
MIG				
<15.5	70 (30.7)	44 (19.3)	1	0.013
15.5-23.3	57 (25.0)	58 (25.4)	0.54 (0.31-0.95), 0.032	
23.3-36.8	60 (26.3)	53 (23.2)	0.71 (0.39-1.27), 0.247	
>36.8	41 (18.0)	73 (32.0)	0.40 (0.22-0.74), 0.003	
IL-10				
Undetectable	191 (83.8)	171 (75.0)	1	0.021
Detectable	37 (16.2)	57 (25.0)	0.58 (0.37-0.92)	
IL1-beta				
Undetectable	164 (71.9)	177 (77.6)	1	0.160
Detectable	64 (28.1)	51 (22.4)	1.35 (0.89-2.07)	
IL-1a				
Undetectable	188 (82.5)	177 (77.6)	1	0.200
Detectable	40 (17.5)	51 (22.4)	0.74 (0.47-1.17)	
TGF-a				
Undetectable	188 (82.5)	177 (77.6)	1	0.200
Detectable	40 (17.5)	51 (22.4)	0.74 (0.47-1.17)	
CRP				
<396240.6	59 (25.9)	55 (24.1)	1	0.400
396240.6-913763.3	62 (27.2)	52 (22.8)	1.16 (0.67-2.01), 0.590	
913763.3-1906040.9	51 (22.4)	63 (27.6)	0.75 (0.44-1.28), 0.290	
>1906040.9	56 (24.6)	58 (25.4)	0.96 (0.54-1.73), 0.890	
IFN-r				
<1.0	135 (59.2)	127 (55.7)	1	0.130
>1.0	93 (40.8)	101 (44.3)	0.95 (0.90-1.01)	
IL-6				
<5.3	103 (45.2)	78 (34.2)	1	0.690
5.3-14.1	37 (16.2)	99 (43.4)	0.22 (0.12-0.39), 0.001	
>14.1	88 (38.6)	51 (22.4)	0.98 (0.61-1.58), 0.940	

Abbreviations: N (%), number (percentage); OR, odds ration; 95% CI, 95% confidence interval. Adjusted for matching variables (age, sex and smoking history), history of chronic bronchitis/emphysema, history of coronary heart disease or heart attack, family history of lung cancer and regular use of aspirin/ibuprofen.

Supplementary Table S3: Pearson correlation analysis of MDC and BLC

Biomarkers	Mean (SD), pg/mL	Correlation Coefficient	P value
BLC	47.6 (38.2)	-0.022	0.645
MDC	122.9 (101.9)		

Abbreviations: SD, standard deviation.

Supplementary Table S4: The diagnostic ability of BLC for subcentimeter lung cancer

Biomarker, pg/mL	Patients N (%)	Controls N (%)	Sensitivity (95% CI)	Specificity (95% CI)	Positive LR (95% CI)	AUC (95% CI)	P
BLC							
>36.0	51 (71.8)	34 (47.9)	0.72 (0.60-0.82)	0.52 (0.40-0.64)	1.50 (1.10-2.05)	0.62 (0.53-0.71)	0.014
<36.0	20 (28.2)	37 (52.1)					

Abbreviations: Positive LR, positive likelihood ratio; 95% CI, 95% confidence interval; AUC, area under the curve.

Supplementary Table S5: The different levels of inflammatory biomarkers among patients with AIS, MIA and IA

Biomarkers	AIS (pg/mL), mean (SD)	MIA (pg/mL), mean (SD)	IA (pg/mL), mean (SD)	P_1	P_2	P_{trend}
BLC	28.4±3.3	52.9±35.3	56.3±32.6	0.008	0.720	0.046
MDC	70.8±40.4	88.5±53.0	94.2±47.4	0.340	0.670	0.850
MIG	21.7±13.9	18.5±8.6	28.2±17.0	0.530	0.100	0.250

Abbreviations: AIS, adenocarcinoma in situ; MIA, minimally invasive adenocarcinoma; IA, invasive adenocarcinoma; SD, standard deviation. P_1: MIA vs. AIS; P_2: IA vs. MIA.
